# Real-time monitoring of spinal cord hemodynamics with laser speckle contrast imaging during pedicle subtraction osteotomy in rabbits

**DOI:** 10.3389/fsurg.2025.1578420

**Published:** 2025-07-09

**Authors:** Zheng Ren, Jing Wang, Xiaolong Ye, Yuan Ma

**Affiliations:** ^1^ The Sixth School of Clinical Medicine, Xinjiang Medical University, Urumqi, Xinjiang, China; ^2^ Xinjiang Institute of Spinal Surgery, The Sixth Affiliated Hospital of Xinjiang Medical University, Urumqi, Xinjiang, China

**Keywords:** speckle contrast imaging (LSCI), pedicle subtraction osteotomy (PSO), spinal cord hemodynamics, intraoperative blood flow monitoring, animal model

## Abstract

**Objective:**

This study aimed to evaluate the efficacy of Speckle Contrast Imaging (LSCI) in real-time monitoring of spinal cord hemodynamic changes during spinal osteotomy in rabbits and to assess its clinical relevance for intraoperative blood flow management.

**Methods:**

Thirty-one healthy male New Zealand white rabbits (clean-grade) were subjected to Pedicle subtraction osteotomy (PSO) under general anesthesia. The spinal cord and posterior vasculature were surgically exposed, and LSCI was employed to quantify blood flow perfusion (Perfusion Units, PU) and posterior spinal artery diameter (μm) at four critical stages: (1) pre-osteotomy (baseline), (2) post-osteotomy, (3) post-osteotomy site compression, and (4) post-dura removal.

**Results:**

LSCI successfully captured dynamic hemodynamic changes: Blood flow perfusion decreased significantly from baseline (519.22 ± 137.87 PU) to post-osteotomy (315.00 ± 50.24 PU, *P* < 0.05), partially recovered after osteotomy site compression (409.16 ± 55.09 PU, *P* < 0.05), and further declined post-dura removal (237.73 ± 40.46 PU, *P* < 0.05). Arterial diameter expanded from 83.13 ± 14.35 μm (baseline) to 388.53 ± 64.62 μm post-osteotomy (*P* < 0.05), then contracted during compression (269.01 ± 50.48 μm) and dura removal (225.84 ± 50.53 μm, both *P* < 0.05).

**Conclusion:**

LSCI provides reliable, non-invasive real-time monitoring of spinal cord hemodynamics during osteotomy, demonstrating significant perfusion and vascular diameter fluctuations. This technology offers a promising tool for intraoperative spinal cord protection and warrants further clinical translation.

## 1 Introduction

Pedicle subtraction osteotomy (PSO) is a well-established surgical technique for correcting spinal deformities, primarily designed to restore spinal alignment and stabilize global balance (1). By realigning the vertebral column, PSO not only mitigates symptoms of spinal cord and nerve compression, alleviates localized pain, and improves neurological function (2). However, the procedure's inherent manipulation of the spinal cord and adjacent vasculature often induces hemodynamic disturbances. These blood flow alterations may precipitate spinal cord ischemia or injury (3), potentially compromising postoperative recovery and, in severe cases, leading to permanent neurological deficits (4).

Conventional methods for monitoring spinal cord hemodynamics, such as near-infrared spectroscopy (NIRS) and contrast-enhanced ultrasound (CEUS), exhibit significant limitations in clinical practice. NIRS requires invasive optical sensor placement (5), while CEUS depends on exogenous contrast administration (6). Crucially, both techniques lack real-time monitoring capabilities, limiting their utility in dynamic surgical environments (7). These constraints highlight the need for more advanced, less invasive monitoring solutions.

Laser speckle contrast imaging (LSCI) has recently emerged as a promising non-invasive technology for blood flow assessment, gaining prominence in neurovascular research (8–12). Its applications in spinal cord studies have expanded significantly, encompassing intraoperative blood flow visualization (13) and acute spinal cord injury evaluation (8). A key advantage of LSCI is its ability to generate real-time, high-resolution microvascular perfusion maps without contrast agents (14), offering superior practicality over traditional methods. Clinical studies have further validated its efficacy in tracking spinal cord blood flow changes in both traumatic (8) and surgical settings (13), underscoring its translational potential.

Building on these advancements, this study investigates the use of LSCI in a rabbit PSO model to evaluate its accuracy in monitoring real-time hemodynamic fluctuations. While clinical validation in human patients remains pending, our preclinical study aims to establish LSCI as a reliable research tool for elucidating spinal cord perfusion dynamics during surgery. The technology's non-invasiveness and exceptional temporal resolution (15) could substantially enhance surgical safety by enabling immediate detection of perfusion deficits. However, its definitive clinical utility requires confirmation through human trials.

By providing foundational insights into spinal cord hemodynamics, this research seeks to bridge the gap between experimental studies and clinical implementation, ultimately facilitating the adoption of LSCI in PSO and related spinal surgical procedures.

## 2 Materials and methods

### 2.1 Experimental animals

Thirty-one healthy male New Zealand white rabbits [SYSK (Xin)-2024-0020; weight: 2.53 ± 0. 07 kg; age: 6.6 ± 0.54 weeks] were procured from the Animal Experiment Center of Xinjiang Medical University [license: SCXK (Xin) 2023-0001]. The animals were housed under standardized conditions (22 ± 1°C, 55 ± 5% humidity, 12-hour light/dark cycle) with *ad libitum* access to food and water. All experimental protocols were reviewed and approved by the Ethics Committee of the Sixth Affiliated Hospital of Xinjiang Medical University (Approval No.: LFYLLSC20241015-02) in compliance with institutional guidelines for animal welfare.

### 2.2 Experimental materials

The surgical suite was equipped with a temperature-controlled small animal operating table, precision microsurgical instruments (including Dumont forceps and Vannas scissors), and specialized spinal osteotomy tools (e.g., Kerrison rongeurs and micro-scalpels). Spinal cord hemodynamics were monitored in real-time using an LSCI system (RFLSI III, Shenzhen Ruiwo De) integrated with intraoperative fluoroscopic guidance (C-arm system, 70 kVp, 2 mA).

To ensure measurement accuracy, the LSCI system underwent:Daily calibration using a reflectance phantom (20% reflectivity, 5 repeated measurements);Weekly validation against a flow phantom (0–1,000 perfusion units, linearity *R*^2^ > 0.99).

Three consecutive measurements were acquired at each time point, and the mean value was calculated to reduce temporal variability.

### 2.3 Anesthesia protocol

General anesthesia was induced via slow intravenous injection of propofol (2–2.5 mg/kg) administered through the marginal ear vein, followed by local infiltration of lidocaine (2%, 1–2 ml) at the surgical site. Anesthetic depth was confirmed by the absence of corneal and pedal reflexes. Throughout the procedure, sterile physiological saline (0.9% NaCl, 500 ml) was delivered intravenously at 10 ml/kg/h to maintain hydration. Vital parameters (SpO₂, heart rate, and respiratory rate) were continuously monitored using a multiparameter veterinary monitor.

### 2.4 Surgical procedure

**Figure 1:**
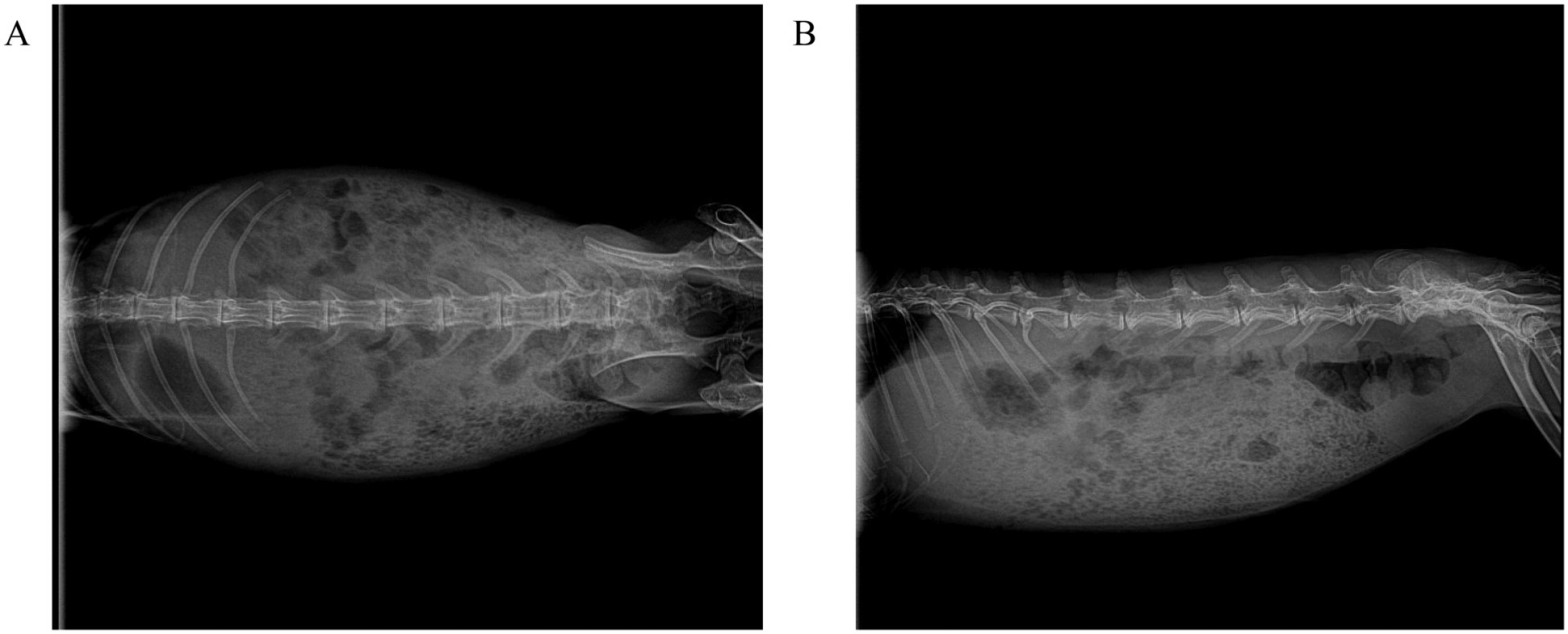
Standard radiographic anatomical reference of the spine in experimental New Zealand white rabbits. **(A)** Anteroposterior (AP) projection: demonstrates bilateral symmetry of vertebral bodies, integrity of costovertebral joints, and pedicle projection, serving as a baseline for assessing scoliosis. **(B)** Lateral projection: clearly depicts physiological thoracic kyphosis and lumbar lordosis, with maintained intervertebral disc space height and no signs of osteoproliferation or bone destruction.

After a 12-hour fasting period, rabbits were prepared for surgery following standard protocols, including hair removal, antiseptic scrubbing, and anesthesia induction. Baseline spinal anatomy was verified using anteroposterior and lateral radiographic imaging (Figures 1A,B).

**Figure 2:**
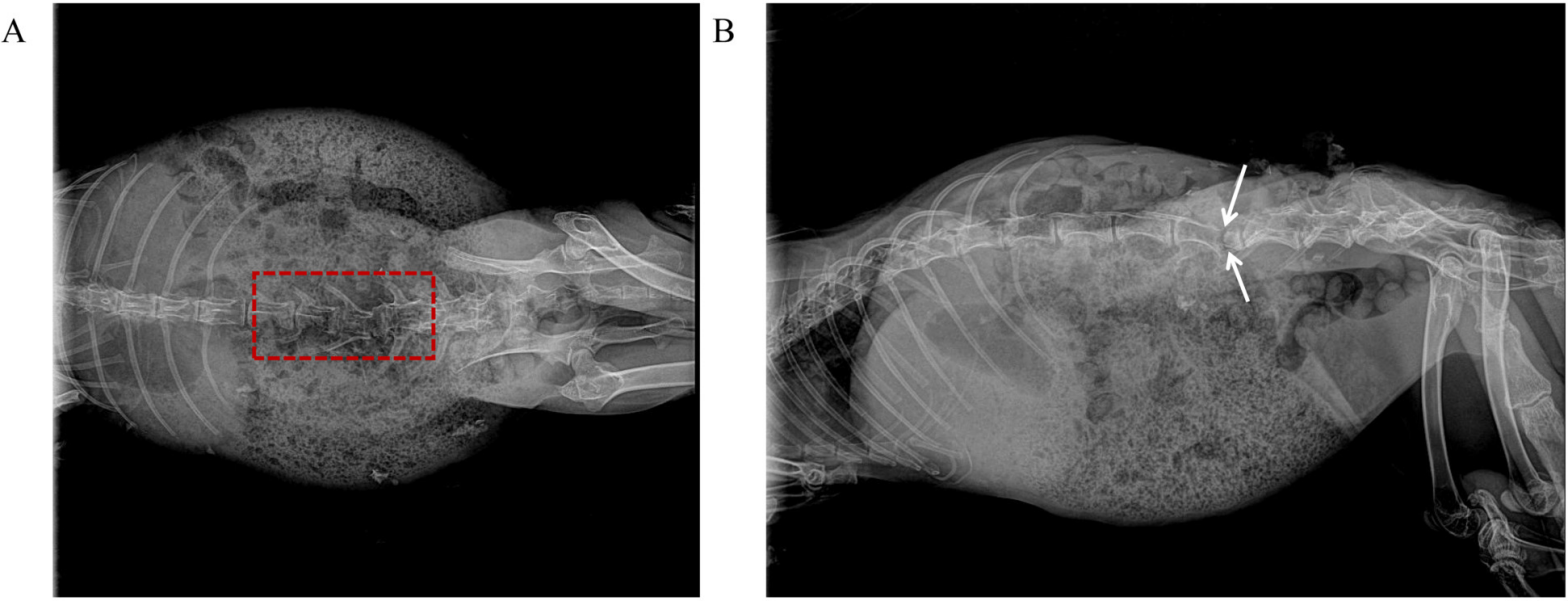
Intraoperative fluoroscopic images of PSO at L4–L5 in a rabbit model. **(A)** Anteroposterior view demonstrates completed 360° circumferential osteotomy of L4 vertebral body (dashed red box indicates osteotomy area, with intact medial pedicle wall preserved). **(B)** Lateral view confirms completion of anterior column wedge osteotomy (white double-headed arrows indicate osteotomy extent, approximately 30° wedge angle, with intact posterior wall cortex).

With the animals in a prone position, a midline incision was made to expose the L1–L7 vertebrae. The spinous processes and laminae were resected to visualize the spinal cord vasculature. Pedicle screws were inserted under fluoroscopic guidance, followed by bilateral 360° osteotomies (Figures 2A,B). LSCI data were collected at four predefined surgical stages: (1) pre-osteotomy (baseline), (2) post-osteotomy, (3) post-osteotomy site compression, and (4) post-dura removal.

**Figure 3:**
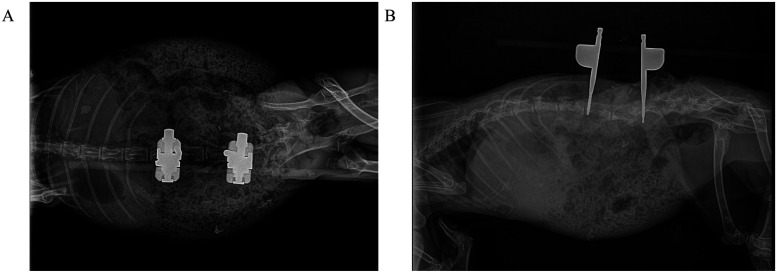
Postoperative radiographic assessment of L4–L5 osteotomy site compression in rabbits. **(A)** Anteroposterior view demonstrates precise cortical apposition at the compressed osteotomy site with optimal screw trajectory. **(B)** Lateral view confirms maintained vertebral height reduction and physiological lordotic alignment restoration.

Postoperative radiographs confirmed appropriate implant positioning (Figures 3A,B). Euthanasia was performed in compliance with AVMA guidelines using an intravenous overdose of pentobarbital sodium (150 mg/kg).

### 2.5 LSCI data acquisition

An LSCI system (780 nm wavelength, 5 ms exposure time, 30 Hz frame rate) was employed to acquire speckle images, which were subsequently processed into high-resolution perfusion maps (1,280 × 960 pixels). Two blinded, independent analysts quantified perfusion units (PU) and vessel diameters, with no knowledge of the surgical phases. Triplicate measurements were averaged for each time point, and the system was recalibrated between sessions following standardized protocols to ensure measurement consistency.

### 2.6 Blinding protocol

To minimize bias, the lead surgeon performed all procedures without access to intraoperative LSCI data, while a dedicated technician operated the imaging system. Final data analysis was conducted by an independent researcher to ensure complete blinding.

### 2.7 Statistical analysis

Continuous data are expressed as mean ± standard deviation (SD). Within-group comparisons were analyzed using paired Student's *t*-tests (SPSS 20.0, IBM Corp.), with statistical significance defined as *P* < 0.05.

## 3 Results

### 3.1 LSCI-Based hemodynamic assessment

**Figure 4:**
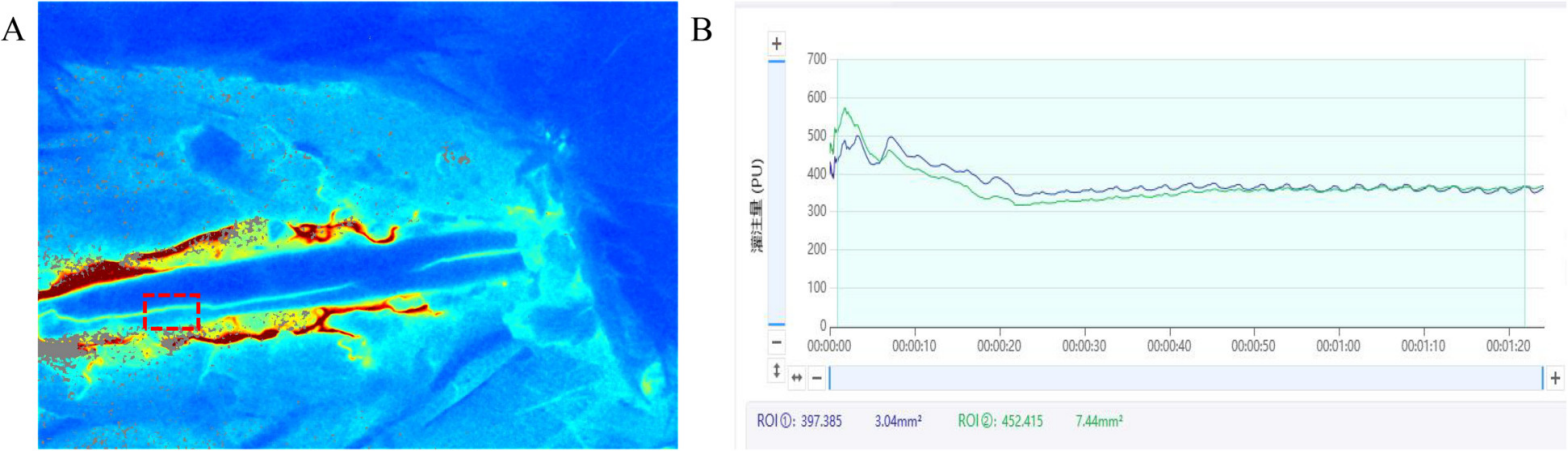
Intraoperative LSCI blood flow real-time monitoring system interface in a rabbit PSO model. This figure illustrates the LSCI interface for real-time hemodynamic monitoring during rabbit laminectomy and PSO. The system interface primarily includes: **(A)** real-time blood flow perfusion pseudocolor map: displays dynamic blood flow fluctuations in the posterior spinal artery region using a color gradient (e.g., red-yellow-blue). **(B)** Dynamic blood flow fluctuation curve (upper right): high-frequency sampling at 500 Hz, reflecting instantaneous blood flow changes in the selected region of interest (ROI) within the surgical field. Quantitative analysis panel (lower right): shows parameters such as mean perfusion units (PU) and relative blood flow change rate, with the ROI position marked by a dashed box.

In the present study, laminectomy was initially performed to expose the spinal cord in a rabbit model of PSO. Spinal cord hemodynamics were subsequently evaluated using LSCI, a non-invasive optical technique that enables high-resolution visualization of vascular architecture through quantitative analysis of speckle contrast patterns. This approach integrates both spatial and temporal variations in red blood cell velocity and concentration, thereby permitting dynamic, indirect quantification of microvascular perfusion and vascular diameter changes (Figure 4).

**Figure 5:**
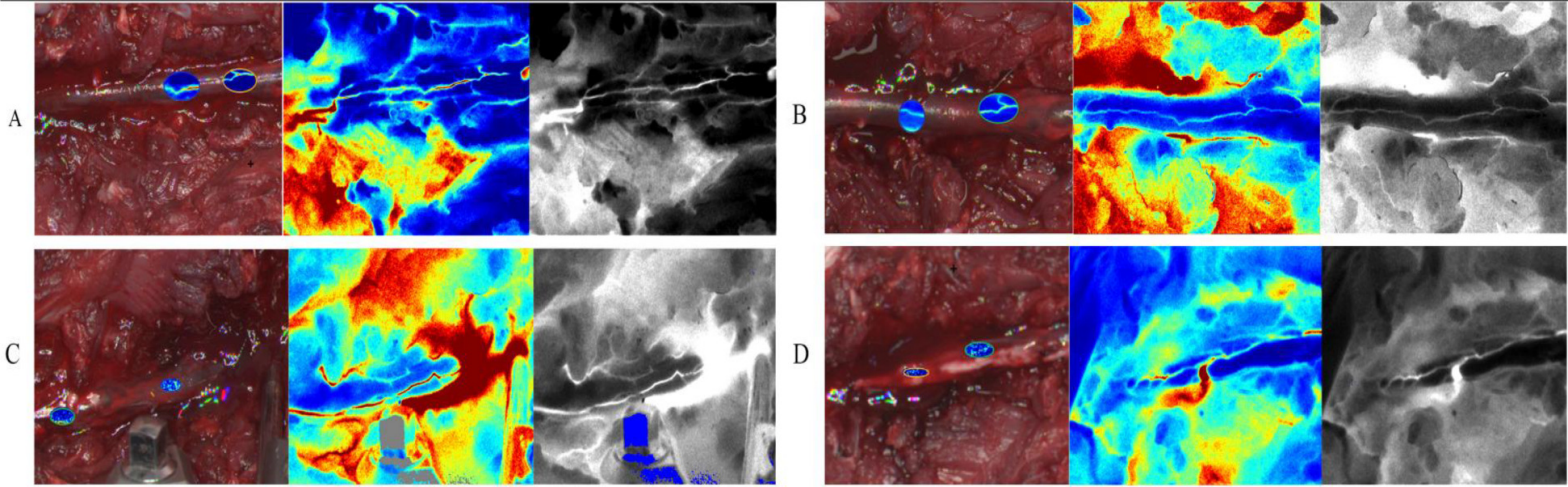
Dynamic changes and blood flow perfusion characteristics of the rabbit posterior spinal artery during different stages of PSO (LSCI). **(A)** Pre-osteotomy (baseline): anatomical positioning and baseline perfusion of the posterior spinal artery (Bright-field: anatomical structure; pseudocolor: velocity distribution; Grayscale: perfusion signal intensity); **(B)** post-osteotomy (10 min post-op): exposure range of the posterior spinal artery and acute-phase perfusion changes following laminectomy; **(C)** osteotomy site compression (20 min post-op): immediate regulatory effects on vascular trajectory and regional hemodynamics after spinal axial shortening; **(D)** dura removal (30 min post-op): terminal perfusion pattern and vessel-tissue interface characteristics with direct vascular exposure.(Imaging modalities: Bright-field - anatomical reference; pseudocolor - velocity quantification; Grayscale - signal intensity analysis; intervals: 10 min between stages).

Preoperative LSCI analysis revealed intact posterior spinal arteries with physiologically normal blood flow patterns, as demonstrated in Figure 5A. In contrast, postoperative pseudo-color LSCI images (Figures 5B–D) exhibited significant hemodynamic alterations, characterized by: (1) a pronounced reduction in perfusion intensity, as indicated by the transition to lighter pseudo-coloration; and (2) concurrent vascular dilation, suggesting compensatory vasodilation of the posterior spinal artery following PSO. These morphological and functional changes collectively indicate substantial microcirculatory disturbances induced by the surgical intervention.

### 3.2 Hemodynamic changes in the posterior spinal artery during PSO

Quantitative assessment of spinal cord perfusion demonstrated significant hemodynamic alterations throughout the surgical procedure. Baseline LSCI measurements prior to osteotomy (pre-PSO) revealed a mean blood flow of 519.22 ± 137.87 PU. Following osteotomy completion (post-PSO), perfusion values decreased significantly to 315.00 ± 50.24 PU (*P* < 0.05), representing a 39.33% reduction from baseline.

Subsequent osteotomy site compression resulted in partial hemodynamic recovery, with perfusion increasing to 409.16 ± 55.09 PU. This constituted a 21.20% reduction from baseline, but reflected a 29.90% improvement compared to immediate post-PSO values. The final surgical phase involving post-dura removal produced the most pronounced perfusion impairment, with values declining to 237.73 ± 40.46 PU—a 54.21% reduction from baseline and a 41.90% decrease relative to post-shortening measurements.

Comparative analysis revealed all perfusion changes reached statistical significance (*P* < 0.05). Notably, the observed 24.53% reduction between osteotomy site compression and post-dura removal phases highlights the critical impact of dural integrity on spinal cord perfusion (Table 1). These findings confirm LSCI's clinical utility in detecting real-time hemodynamic fluctuations during PSO procedures.

**Table 1:** Quantitative LSCI analysis of blood flow perfusion (PU) changes across surgical phases of PSO in rabbit model (*n* = 31).

Pair	Comparison	Mean difference ± SD	SE	95% CI	*t*	df	*p*
1	Pre-osteotomy vs. post-osteotomy	204.02 ± 153.23	27.52	[147.81, 260.22]	7.413	30	<.001*
2	Pre-osteotomy vs. osteotomy site compression	110.06 ± 149.06	26.77	[55.39, 164.74]	4.111	30	<.001*
3	Pre-osteotomy vs. dura removal	281.49 ± 149.66	26.88	[226.60, 336.39]	10.472	30	<.001*
4	Post-osteotomy vs. osteotomy site compression	−93.95 ± 75.28	13.52	[−121.57, −66.34]	−6.949	30	<.001*
5	Post-osteotomy vs. dura removal	77.47 ± 52.82	9.49	[58.10, 96.85]	8.167	30	<.001*
6	Osteotomy site compression vs. dura removal	171.43 ± 72.16	12.96	[144.96, 197.90]	13.227	30	<.001*

Data presented as mean difference ± standard deviation. All comparisons were analyzed by paired *t*-tests with Bonferroni correction (*α* = 0.0083). Negative values indicate reduced perfusion relative to the reference stage. SE, standard error; CI, confidence interval; df, degrees of freedom; PSO, pedicle subtraction osteotomy.

*Indicates statistically significant differences (*p* < 0.001).

### 3.3 Diametric variations of the posterior spinal artery during PSO procedures

The posterior spinal artery exhibited progressive diametric changes across surgical phases. At pre-osteotomy baseline, the arterial diameter measured 83.13 ± 14.35 μm. Following osteotomy completion, significant vasodilation occurred, with the diameter expanding to 388.53 ± 64.62 μm, representing a 3.67-fold increase from baseline. Subsequent osteotomy site compression resulted in a measurable reduction to 269.01 ± 50.48 μm (2.24-fold baseline), while final dural removal further decreased the diameter to 225.84 ± 50.53 μm (1.72-fold baseline).

Comparative analysis revealed a 30.76% diameter reduction from post-osteotomy values after osteotomy site compression, with an additional 41.87% decrease following dural removal. The post-dural removal measurements were 16.05% smaller than osteotomy site compression. All observed diametric changes reached statistical significance (*P* < 0.05) (Table 2), confirming LSCI as an effective modality for intraoperative vascular monitoring during PSO procedures.

**Table 2:** Quantitative LSCI analysis of posterior spinal artery diameter (μm) changes across surgical phases of PSO in rabbit model (*n* = 31).

Pair	Comparison	Mean difference ± SD	SE	95% CI	*t*	df	*p*
1	Pre-osteotomy vs. post-osteotomy	−305.40 ± 70.80	12.72	[−331.37, −279.43]	−24.018	30	<.001*
2	Pre-osteotomy vs. osteotomy site compression	−185.88 ± 53.09	9.53	[−205.35, −166.41]	−19.496	30	<.001*
3	Pre-osteotomy vs. dura removal	−142.71 ± 52.53	9.43	[−161.98, −123.44]	−15.125	30	<.001*
4	Post-osteotomy vs. osteotomy site compression	119.52 ± 71.00	12.75	[93.48, 145.56]	9.373	30	<.001*
5	Post-osteotomy vs. dura removal	162.69 ± 78.38	14.08	[133.94, 191.44]	11.557	30	<.001*
6	Osteotomy site compression vs. dura removal	43.17 ± 81.19	14.58	[13.39, 72.96]	2.961	30	<.001*

Data are presented as mean difference ± standard deviation. All comparisons were analyzed by paired t-tests with Bonferroni correction (*α* = 0.0083). Negative values indicate diameter reduction.

*Indicates statistically significant differences (*p* < 0.001).

## 4 Discussion

Spinal deformity, as a complex three-dimensional displacement disorder, presents significant therapeutic challenges. Epidemiological data indicate that the number of spinal deformity patients in China has exceeded 3 million, with adult cases increasing annually due to population aging. Adolescent idiopathic scoliosis has become the third most prevalent condition affecting youth health (16). Complex spinal deformities, often involving lumbosacral or cervicothoracic regions with double major curves, kyphoscoliosis, and severe vertebral rotation, are characterized by extreme anatomical distortions. These deformities lead to profound cosmetic disfigurement, cardiopulmonary compromise, neurological deficits, psychological distress, reduced life expectancy, and high disability rates, imposing substantial socioeconomic burdens.

PSO remains the gold standard for correcting severe deformities (17). However, this procedure carries considerable risks, with neurological complication rates as high as 21.2% (18), underscoring the critical need for intraoperative spinal cord monitoring. During PSO, surgical maneuvers including pedicle screw placement, osteotomy execution, and rod rotation can alter spinal cord morphology, potentially inducing ischemia, hypoxia, and oxidative stress. The spinal cord's microvasculature demonstrates remarkable sensitivity to these pathological changes (19), making hemodynamic parameters excellent biomarkers for ischemic injury. Accumulating evidence confirms that post-injury hemodynamic alterations significantly impact neurological outcomes (20). Tang (21) further established that spinal blood flow dynamics reflect regulatory states of spinal neural circuits, validating the existence of neurovascular coupling mechanisms in the spinal cord.

Currently, the primary modalities for spinal cord hemodynamic imaging encompass two-photon microscopy, arterial spin labeling (ASL), contrast-enhanced ultrasound (CEUS), near-infrared diffuse reflectance spectroscopy (NIR-DRS), fluorescence microscopy, total internal reflection fluorescence (TIRF), and light-sheet fluorescence microscopy (LSFM) (22–24). While these techniques provide valuable hemodynamic information, they are constrained by several limitations, including prolonged acquisition times and suboptimal spatiotemporal resolution. In contrast, optical imaging modalities offer distinct advantages in terms of rapid data acquisition, continuous monitoring capability, portability, and cost-effectiveness, making them promising alternatives for hemodynamic assessment.

LSCI has emerged as a particularly advantageous optical technique due to its non-contact operation, absence of ionizing radiation, cost-efficiency, and real-time imaging capability. The fundamental principle of LSCI involves illuminating biological tissue with coherent laser light, generating dynamic speckle patterns that are subsequently analyzed through pseudo-coloring and speckle contrast computation. This process enables visualization of vascular morphology and two-dimensional blood flow distribution (14). The strength of LSCI lies in its quantitative analysis of speckle pattern statistics, which provides accurate estimation of microvascular perfusion and blood flow dynamics. This technique has demonstrated significant clinical utility across various applications, including burn wound assessment, retinal perfusion mapping, cerebral blood flow monitoring, and microvascular imaging of cutaneous, hepatic, esophageal, and colonic tissues (25).

Our research represents the first application of LSCI in a PSO animal model, addressing a critical gap in current clinical imaging approaches. The non-invasive nature, rapid acquisition, and quantitative capabilities of LSCI position it as an ideal modality for investigating the complex hemodynamic changes following spinal cord injury. The technique's proven efficacy in both preclinical and clinical settings, particularly in neurological and microvascular applications, underscores its potential for translational research in spinal cord injury management.Mechanistically, our rabbit PSO model revealed operation-specific impacts on spinal cord perfusion: osteotomy, osteotomy site compression, and dural removal caused 39.3%, 21.2%, and 54.2% reductions in blood flow, respectively. These quantitative findings not only confirm the crucial role of dural integrity in maintaining spinal perfusion but also provide empirical evidence for optimizing surgical sequences. Notably, the 29.9% perfusion recovery during osteotomy site compression maneuvers supports the “spinal cord tension-blood flow” theory proposed by Li (26) and Kawahara (27), validating the clinical practice of staged osteotomy site compression techniques. Complementary findings by Lu QA (28) demonstrated that incremental osteotomy site compression (1/4 and 2/4 length reductions) effectively decreases cord tension, creating essential buffer space for vascular and neural structures during deformity correction.

Compared with existing monitoring technologies, LSCI offers distinct advantages: (1) true real-time wide-field imaging (vs. single-point sampling in laser Doppler); (2) contrast-agent-free operation (eliminating allergic risks); (3) cost-effectiveness (approximately 10% of intraoperative MRI expenses). These features make LSCI particularly suitable for widespread hospital adoption, potentially revolutionizing the current experience-dependent monitoring paradigm in PSO surgeries.

While LSCI holds significant promise for intraoperative spinal cord perfusion monitoring, several critical challenges must be addressed to facilitate its clinical translation. From a technical perspective, the unique anatomical characteristics of the human spine—such as variations in lamina thickness and epidural fat distribution—necessitate optimization of LSCI parameters, including imaging depth (15–20 mm) and field of view (≥2 vertebral segments), to ensure reliable signal acquisition. Drawing from established techniques in neurosurgical fluorescence angiography, one potential solution involves developing specialized spinal retractors with integrated LSCI probes, enabling real-time, noninvasive perfusion assessment during surgery. Building upon these technical adaptations, a structured clinical validation pathway is essential: initial cadaveric studies to correlate LSCI-derived perfusion metrics with pressure parameters, followed by safety evaluations in non-deformity decompression procedures, and ultimately, prospective studies in PSO cohorts to assess predictive accuracy for neurological complications. Furthermore, standardized decision-support protocols must be developed, incorporating dynamic alarm thresholds (e.g., a >40% flow reduction persisting for 5 min triggering intervention), real-time trend analysis algorithms, and multimodal integration with somatosensory evoked potentials and other monitoring modalities. Only through this comprehensive, stepwise approach can LSCI evolve from an experimental tool into a clinically viable technology, enhancing precision in spinal surgical care.

Current technical limitations include restricted laser penetration (particularly in obese patients), blood artifact interference, and real-time processing requirements (>30 fps). Solutions may involve 785 nm near-infrared lasers for enhanced tissue penetration and deep learning algorithms for discriminating true perfusion from bleeding artifacts.

Future research should prioritize: (1) establishing Chinese population-specific spinal perfusion databases, (2) developing intelligent early-warning systems, and (3) exploring LSCI's value across spinal procedures. Through 5–8 years of technological refinement and clinical validation, LSCI could potentially establish itself as a standard PSO monitoring tool, reducing neurological complications below 5%. This advancement would not only improve surgical safety but also facilitate a paradigm shift from morphological correction to functional preservation in deformity management.

In conclusion, our LSCI application in PSO demonstrates remarkable potential for real-time spinal cord perfusion monitoring. By providing surgeons with immediate, accurate hemodynamic feedback, this technology enables data-driven decision-making to enhance procedural safety and reduce complications. More importantly, it pioneers a non-invasive monitoring approach for spinal surgery. With continued technological progress and clinical investigation, LSCI may emerge as an indispensable intraoperative tool, propelling the field toward safer and more precise surgical practices.

## Data Availability

The raw data supporting the conclusions of this article will be made available by the authors, without undue reservation.

## References

[1] Zhao Z Bi N Li T Shi Z Xia G Zhang Y Spinal-shortening process positively improves associated syringomyelia in patients with scoliosis after single-stage spinal correction. World Neurosurg. (2021) 152:e161–7. 10.1016/j.wneu.2021.05.073 34052457

[2] Aldave G Hansen D Hwang SW Moreno A Briceño V Jea A. Spinal column shortening for tethered cord syndrome associated with myelomeningocele, lumbosacral lipoma, and lipomyelomeningocele in children and young adults. J Neurosurg Pediatr. (2017) 19(6):703–10. 10.3171/2017.1.PEDS16533 28362188

[3] Riley MS Lenke LG Chapman TM Jr Sides BA Blanke KM Kelly MP. Clinical and radiographic outcomes after posterior vertebral column resection for severe spinal deformity with five-year follow-up. J Bone Joint Surg Am. (2018) 100(5):396–405. 10.2106/JBJS.17.00597 29509617

[4] Yang JL Huang ZF Yin JQ Deng YL Xie XB Li FB A proposed classification system for guiding surgical strategy in cases of severe spinal deformity based on spinal cord function. Eur Spine J. (2016) 25(6):1821–9. 10.1007/s00586-015-4367-2 26769035

[5] Cheung A Tu L Manouchehri N Kim KT So K Webster M Continuous optical monitoring of spinal cord oxygenation and hemodynamics during the first seven days post-injury in a porcine model of acute spinal cord injury. J Neurotrauma. (2020) 37(21):2292–301. 10.1089/neu.2020.7086 32689879

[6] Bruce M DeWees D Harmon JN Cates L Khaing ZZ Hofstetter CP. Blood flow changes associated with spinal cord injury assessed by non-linear Doppler contrast-enhanced ultrasound. Ultrasound Med Biol. (2022) 48(8):1410–9. 10.1016/j.ultrasmedbio.2022.03.004 35523621 PMC9704544

[7] Khaing ZZ Cates LN Hyde J DeWees DM Hammond R Bruce M Contrast-enhanced ultrasound for assessment of local hemodynamic changes following a rodent contusion spinal cord injury. Mil Med. (2020) 185(Suppl 1):470–5. 10.1093/milmed/usz296 32074323

[8] Gallagher MJ Hogg FRA Zoumprouli A Papadopoulos MC Saadoun S. Spinal cord blood flow in patients with acute spinal cord injuries. J Neurotrauma. (2019) 36(6):919–29. 10.1089/neu.2018.5961 30351245

[9] Pion E Asam C Feder AL Felthaus O Heidekrueger PI Prantl L Laser speckle contrast analysis (LASCA) technology for the semiquantitative measurement of angiogenesis in in-ovo-tumor-model. Microvasc Res. (2021) 133:104072. 10.1016/j.mvr.2020.104072 32949573

[10] Tao S Zhang T Zhou K Liu X Feng Y Zhao W Intraoperative monitoring cerebral blood flow during the treatment of brain arteriovenous malformations in hybrid operating room by Laser speckle contrast imaging. Front Surg. (2022) 9:855397. 10.3389/fsurg.2022.855397 35599788 PMC9120635

[11] Vinnett A Kandukuri J Le C Cho KA Sinha A Asanad S Dynamic alterations in blood flow in glaucoma measured with laser speckle contrast imaging. Ophthalmol Glaucoma. (2022) 5(3):250–61. 10.1016/j.ogla.2021.10.005 34673279 PMC9013729

[12] Heeman W Wildeboer ACL Al-Taher M Calon JEM Stassen LPS Diana M Experimental evaluation of laparoscopic laser speckle contrast imaging to visualize perfusion deficits during intestinal surgery. Surg Endosc. (2023) 37(2):950–7. 10.1007/s00464-022-09536-9 36068388 PMC9944003

[13] Miller DR Ashour R Sullender CT Dunn AK. Continuous blood flow visualization with laser speckle contrast imaging during neurovascular surgery. Neurophotonics. (2022) 9(2):021908. 10.1117/1.NPh.9.2.021908 35265733 PMC8900813

[14] Aminfar A Davoodzadeh N Aguilar G Princevac M. Application of optical flow algorithms to laser speckle imaging. Microvasc Res. (2019) 122:52–9. 10.1016/j.mvr.2018.11.001 30414869

[15] Liu C Kılıç K Erdener SE Boas DA Postnov DD. Choosing a model for laser speckle contrast imaging. Biomed Opt Express. (2021) 12(6):3571–83. Published 2021 May 21. 10.1364/BOE.426521 34221679 PMC8221943

[16] Fehlings MG Tetreault L Nater A Choma T Harrop J Mroz T The aging of the global population: the changing epidemiology of disease and spinal disorders. Neurosurgery. (2015) 77(Suppl 4):S1–5. 10.1227/NEU.0000000000000953 26378347

[17] Schwab F Blondel B Chay E Demakakos J Lenke L Tropiano P The comprehensive anatomical spinal osteotomy classification. Neurosurgery. (2014) 74(1):112–20. 10.1227/NEU.0000000000000182o 24356197

[18] Xia L Li P Wang D Bao D Xu J. Spinal osteotomy techniques in management of severe pediatric spinal deformity and analysis of postoperative complications. Spine (Phila Pa 1976). (2015) 40(5):E286–92. 10.1097/BRS.0000000000000728 25494310

[19] Liu X Gu Y Huang C Zhao M Cheng Y Abu Jawdeh EG Simultaneous measurements of tissue blood flow and oxygenation using a wearable fiber-free optical sensor. J Biomed Opt. (2021) 26(1):012705. 10.1117/1.JBO.26.1.012705 33515216 PMC7846117

[20] Bosmia AN Hogan E Loukas M Tubbs RS Cohen-Gadol AA. Blood supply to the human spinal cord: part I. Anatomy and hemodynamics. Clin Anat. (2015) 28(1):52–64. 10.1002/ca.22281 23813725

[21] Tang S Cuellar CA Song P Islam R Huang C Wen H Changes in spinal cord hemodynamics reflect modulation of spinal network with different parameters of epidural stimulation. Neuroimage. (2020) 221:117183. 10.1016/j.neuroimage.2020.117183 32702485 PMC7802109

[22] Khaing ZZ Cates LN DeWees DM Hannah A Mourad P Bruce M Contrast-enhanced ultrasound to visualize hemodynamic changes after rodent spinal cord injury. J Neurosurg Spine. (2018) 29(3):306–13. 10.3171/2018.1.SPINE171202 29905521

[23] Mainard N Tsiakaka O Li S Denoulet J Messaoudene K Vialle R Intraoperative optical monitoring of spinal cord hemodynamics using multiwavelength imaging system. Sensors (Basel). (2022) 22(10):3840. 10.3390/s22103840 35632249 PMC9146887

[24] Behem CR Friedheim T Wipper SH Pinnschmidt HO Graessler MF Gaeth C Real-time assessment of spinal cord microperfusion in a porcine model of ischemia/reperfusion. J Vis Exp. (2020) (166):e62047. 10.3791/62047 33369603

[25] Heeman W Steenbergen W van Dam G Boerma EC. Clinical applications of laser speckle contrast imaging: a review. J Biomed Opt. (2019) 24(8):1–11. 10.1117/1.JBO.24.8.080901 31385481 PMC6983474

[26] Li T Zhao Z Wang Y Xie J Zhang Y Bi N A preliminary study of spinal cord blood flow during PVCR with spinal column shortening: a prospective clinic study in severe rigid scoliokyphosis patients. Medicine (Baltimore). (2020) 99(32):e21579. 10.1097/MD.0000000000021579 32769906 PMC7593061

[27] Kawahara N Tomita K Kobayashi T Abdel-Wanis ME Murakami H Akamaru T. Influence of acute shortening on the spinal cord: an experimental study. Spine (Phila Pa 1976). (2005) 30(6):613–20. 10.1097/01.brs.0000155407.87439.a2 15770174

[28] Lu QA Wang YS Xie JM Li T Shi ZY Du ZS Effect of spinal shortening for protection of spinal cord function in canines with spinal cord angulation. Med Sci Monit. (2019) 25:9192–9. 10.12659/MSM.919313 31791038 PMC6909917

